# The Effect of Dietary Replacement of Ordinary Rice with Red Yeast Rice on Nutrient Utilization, Enteric Methane Emission and Rumen Archaeal Diversity in Goats

**DOI:** 10.1371/journal.pone.0160198

**Published:** 2016-07-28

**Authors:** L. Z. Wang, M. L. Zhou, J. W. Wang, D. Wu, T. Yan

**Affiliations:** 1 Animal Nutrition Institute, Sichuan Agricultural University, Ya'an, China; 2 Agri-Food and Biosciences Institute, Hillsborough, Co Down, United Kingdom; Hellas, GREECE

## Abstract

Twenty castrated Boer crossbred goats were used in the present study with two treatments to examine the effect of dietary replacement of ordinary rice with red yeast rice on nutrient utilization, enteric methane emission and ruminal archaea structure and composition. Two treatment diets contained (DM basis) 70.0% of forage, 21.8% of concentrates and 8.2% of either ordinary rice (control) or red yeast rice (RYR). Nutrient utilization was measured and enteric methane emissions were determined in respiration chambers. Results showed that RYR had significantly lower digestibility of N and organic matter compared to control group. However, feeding red yeast rice did not affect N retention as g/d or a proportion of N intake, and reduced heat production as MJ/d or as a proportion of metabolizable energy intake, thus leading to a higher proportion of metabolizable energy intake to be retained in body tissue. RYR also had significantly lower methane emissions either as g/d, or as a proportion of feed intake. Although feeding red yeast rice had no negative effect on any rumen fermentation variables, it decreased serum contents of total cholesterol, triglycerides, HDL-cholesterol and LDL-cholesterol. In the present study, 75616 archaeal sequences were generated and clustered into 2364 Operational Taxonomic Units. At the genus level, the predominant archaea in the rumen of goats was *Methanobrevibacter*, which was significantly inhibited with the supplementation of red yeast rice. In conclusion, red yeast rice is a potential feed ingredient for mitigation of enteric methane emissions of goats. However, caution should be taken when it is used because it may inhibit the digestibility of some nutrients. Further studies are required to evaluate its potential with different diets and animal species, as well as its effects on animal health and food safety.

## Introduction

Methane (CH_4_) is a greenhouse gas and about 25 times effective than carbon dioxide (CO_2_) in the global warming potential [1]. The CH_4_ emission from enteric fermentation in ruminants accounts for about 10% of the total anthropogenic CH_4_ [2]. In addition, about 8 to 14% of the ruminant ingested dietary energy may lose in the manner of CH_4_ emission [3]. Therefore, mitigation of CH_4_ emission from ruminants not only benefits the environment, but also improves the feed efficiency. Different from bacteria and other microorganisms, archaea needs 3-hydroxy-3methyl-glutaryl coenzyme A reductase (HMG-CoA reductase) during the process of cell membrane biosynthesis, which is similar with cholesterol biosynthesis in human. It is expected that use of inhibitor of HMG-CoA reductase can restrict the growth and proliferation of archaea and thus reduce CH_4_ production in the rumen. In the 1970’s, scientists working on cardiovascular diseases found that lovastatin can highly inhibit the activity of HMG-CoA reductase [4]. The previous *in vitro* experiments have indicated that lovastatin can inhibit archaea growth and mitigate the CH_4_ production [5–8]. However, the commercial lovastatin is a prescription medication for human and the approved use as a feed additive for animals has not been published. Furthermore, the lovastatin in the commercial market contains only 2% of active beta-hydroxy acid open ring form (H-form), and the remainder 98% is the inactive gamma-lactone closed ring form (L-form) which has no ability to inhibit the HMG-CoA reductase unless it is hydrolyzed by carboxylesterases *in vivo* to H-form [7].

The red yeast rice, a well-known traditional Chinese food produced by the fermentation of ordinary rice with *Monascus purpureus*, contains monacolin K, a natural form of lovastatin. Most of the lovastatin in red yeast rice is H-form which can directly inhibit archaeal activities in the rumen. Up to date, there have been only few publications [9, 10] for use of red yeast rice as an additive to reduce CH_4_ emissions in sheep and beef cattle with inconclusive results. Further studies are required to evaluate the effect of dietary inclusion of red yeast rice on enteric CH_4_ emissions. Furthermore, it is a prerequisite for the use of feed additives which should have no negative effect on feeding efficiency. However, there is little information available in the literature on the effect of dietary inclusion of red yeast rice on feeding efficiencies, as well as on ruminal archaeal structure and composition. Therefore, the present study was designed to fill this gap of knowledge by evaluating the possible effect of red yeast rice on energy and nitrogen utilization efficiencies, as well on enteric CH_4_ emissions, rumen fermentation and archaeal diversity in the rumen using goats as test animals.

## Material and Methods

### Experimental design and animal management

The experiment protocol was approved by the Animal Care and Ethics Committee of Sichuan Agricultural University (Permit Number: DKY-S20143242). Twenty castrated Boer crossbred goats (Boer × Jianchang Black) were used in the present study with two treatments to examine the effect of dietary replacement of ordinary rice with red yeast rice. Two treatment diets contained (DM basis) 70% of forage, 21.8% of concentrates and 8.2% of either ordinary rice (control, CT) or red yeast rice (red yeast rice, RYR) (Table 1). Animals were 20 months old and had an average body weight of 41.6±2.6 kg at the commencement of the study. The red yeast rice (Jiacheng Medicines and Health Products, Wuhan, China) contained 2 mg/g of lovastatin (DM basis) with 56% in the H-form measured using high-performance liquid chromatography systems (SCL-10A, Shimadzu, Tokyo, Japan) [7]. The goats were housed in individual pens with free access to water. Each goat was offered total mixed ration at a restricted level of 1.1 kg DM/d (Table 1) in two equal amounts at 0900 and 1700 h. After 14 days of adaption, the goats were transferred into metabolism cages to collect feces (antisepticised with 10% formaldehyde, w/w of 3% of feces) and urine (acidified with 10% HCl, v/v of 5% of urine) for 6 days. A daily sample of feces and urine were taken and stored frozen at -20°C for later analysis. Immediately after the completion of measurements in metabolism cages, blood serum samples from each goat were taken 2 h after morning feeding and stored at -20°C until required. Then the goats were weighted and transferred into indirect open-circuit respiration chambers. The animals were housed in chambers for 3 days with oxygen consumption and carbon dioxide and CH_4_ outputs measured during the final 2 days. All procedures, analytical methods, and calculations used in the respiration chambers were as reported previously [11]. Immediately after the completion of chamber measurements, rumen sample was collected before feeding in the morning using a stomach tube attached to an electric pump as described previously [12]. The rumen content was strained through four layers of cheese cloth and the liquid sample was divided into two portions. One was stored at –80°C until the DNA extraction and other stored at –20°C for short chain fatty acids (SCFAs) and ammonia-nitrogen analyses.

**Table 1:** The ingredient composition and chemical contents of diets.

	CT	RYR
Ingredient composition (%, DM basis)		
Alfalfa meal	34.8	34.8
Rice straw	35.2	35.2
Corn	3.3	3.3
Ordinary rice	8.2	0
Red yeast rice	0	8.2
Soybean meal	5.1	5.1
Wheat bran	12.4	12.4
Premix^a^	0.5	0.5
Salt	0.5	0.5
Chemical contents (%, DM basis)		
Metabolizable energy ^b^ (MJ/kg)	8.1	7.9
Crude protein	10.9	11.0
Neutral detergent fiber	49.4	48.6
Acid detergent fiber	29.4	29.0
Calcium	0.99	1.04
Phosphorus	0.33	0.35

^a^Premix provides: Ca (as calcium carbonate) 1.5 g/kg DM; Fe (as ferrous sulfate) 30 mg/kg DM; Cu (as copper sulfate) 10 mg/kg DM; Zn (as zinc sulfate) 50 mg/kg DM; Mn (as manganese sulfate) 60 mg/kg DM; Vitamin A 2937 IU/kg DM; Vitamin D 343 IU/kg DM; Vitamin E 30 IU/kg DM.

^b^Metabolizable energy was calculated using gross energy intake minus energy excretions from feces, urine and methane and then divided by DM intake, with all data measured in metabolizable cages and calorimeter chambers.

### Analysis of samples

Samples of feed and feces were dried in a forced-air oven at 65°C for 48 h for DM measurements, then ground to pass through a 1-mm screen. The nitrogen (N) content in the samples was determined by the Kjeldahl method (AOAC, 1990), organic matter (OM) was measured in a muffle furnace at 550°C for 6 hours (AOAC, 1990), calcium and phosphorus was determined using atomic absorption spectrophotometer (AOAC, 1990). Neutral detergent fiber (NDF) and acid detergent fiber (ADF) were determined using the filter bag technique without sodium sulphite or heat stable amylase, and expressed with residual ash. Urine samples were assayed for N and gross energy (GE) concentrations. The rumen SCFAs were analyzed by gas chromatography (GC-2014FRGA1, Shimadzu, Tokyo, Japan) [13]. Ammonia N concentration in the rumen liquid was measured using a colorimetric technique as described previously [14]. Blood serum content of total cholesterol (TC), triglyceride (TG), high density lipoprotein cholesterol (HDL-C) and low density lipoprotein cholesterol (LDL-C) levels were quantified using a biochemical analyzer (Modular P800, Roche, Mannheim, Germany). The GE contents in feed, feces and urine were determined by an oxygen bomb calorimeter (Model 1281, Parr, Moline, USA). Heat production (HP) was estimated based on the equation: HP (KJ) = 16.1735×O_2_ (L)+5.0208×CO_2_ (L) −2.1673×CH_4_ (L)– 5.9873×Urine nitrogen (g) [15].

### Analysis of rumen arechaeal composition

The total DNAs in the rumen liquid were extracted and purified using the method described previously [16]. The archaea specific primers with barcode, 341F (5’- CCTAYGGGRBGCASCAG -3’) and 806R (5’- GGACTACHVGGGTWTCAAT -3’) [17], were used to amplify archaeal 16S rRNA genes. The amplification was initiated with a denaturation at 94°C for 3 min, followed by 30 cycles of 94°C for 30 s, 58°C for 30 s and 72°C for 90 s, and a last extension at 72°C for 5 min. The total volume of the reaction mixture was 50 μL consisted of 200 nM of each primer, 5 μL of 2.5 mmol/L dNTP mixture, 5 μL of 10×*Ex Taq* buffer (20 mmol/L Mg^2+^; TaKaRa Inc., Dalian, China), 0.35 μg of template DNA, 2 mM of MgCl_2_ and four units of Taq DNA polymerase (Takara Inc., Dalian, China), approximately 37 μL milli-Q water. The amplicons were purified using a PCR Clean-Up system (Promega, Madison, USA) with a purification kit (QIAGEN, Australia), and were quantified using QuantiFlour^™^-ST fluorometer (Promega, Madison, USA). Three replicates of DNA extract from each sample were amplified and were mixed together. The PCR products were sent to Macrogen Inc (Seoul, South Korea) and were throughput sequenced on the Illumina MiSeq 300PE Sequencing Platform.

Pyrosequencing reads were mainly analyzed using QIIME pipeline software [18] in the present study. The poor/low quality sequences including those with uncertain nucleotides, continuous three nucleotides with Q value less than 20, unmatched barcode sequences were discarded. Uchime algorithm [19] implementing in Mothur [20] were used to remove chimeric sequences. Sequencing noise was further reduced using a preclustering methodology [21]. Uclust [22] method was then used to cluster the obtained clean and high quality sequences into operational taxonomic units (OTUs) for eventual taxonomy assignment based on 97% sequence similarity. The most abundant sequence was selected as the representative for each OTU, and was assigned to taxonomic using RDP Classifier [23] (at 80% confidence threshold). The non-archaeal OTUs were removed. Three alpha diversity indices (Shannon-Wiener, Chao1 and Observed-species) were calculated. The weighted Unifrac distance matrix between samples was measured and was visualized using Principal Co-ordinate Analysis (PCoA). The shared archaea were selected using custom Perl scripts. All sequence data in the present study were deposited in the Sequence Read Archive (SRA) of the NCBI database under number PRJNA290544.

### Statistical analysis

The unpaired two-tailed t-test (SPSS Statistics for Windows, Version 22.0, IBM., Armonk, NY, USA) was performed to assess whether there was a significant difference between CT and RYR. Significance level was set at P<0.05.

## Results

### Effect on nutrient digestibility and nitrogen balance

There were no significant differences in DM intake, apparent digestibility of NDF or ADF between the two treatments. The apparent digestibility of N and OM was significantly higher in CT than that in RYR (Table 2). The apparent DM digestibility had a tendency to be lower in RYR compared to CT (P = 0.067).

**Table 2:** The effect of replacement of ordinary rice with red yeast rice on DM intake and apparent nutrient digestibility in goats (n = 20).

	CT	RYR	SED	*P*
Dry matter intake (kg/d)	1.07	1.08	0.01	0.439
Average daily body weight gain (kg/d)	0.18	0.18	0.01	0.775
Nutrient digestibility (%)				
Dry matter	58.1	57.2	0.42	0.067
Organic matter	61.3	56.1	0.50	0.002
Nitrogen	66.4	61.7	0.55	<0.001
Neutral detergent fiber	57.2	58.3	1.93	0.574
Acid detergent fiber	48.1	43.4	5.25	0.400

Diet treatments had no significant effect on N intake, urine N output, or urine N/N intake (Table 3). However, feeding red yeast rice significantly increased fecal N output and fecal N output as a proportion of N intake (P < 0.001), and tended to decrease N retention (P = 0.071) and N retention as a proportion of N intake (P = 0.061).

**Table 3:** The effect of replacement of ordinary rice with red yeast rice on N metabolism in goats (n = 20).

	CT	RYR	SED	*P*
Nitrogen intake and output (g/d)				
Nitrogen intake (NI)	19.1	19.3	0.12	0.709
Fecal nitrogen (FN)	6.4	7.4	0.09	<0.001
Urine nitrogen (UN)	7.8	8.1	0.49	0.580
Retention nitrogen (RN)	4.9	3.8	0.51	0.071
Nitrogen utilization (%)				
FN/NI	33.6	38.3	0.01	<0.001
UN/NI	40.7	41.9	0.03	0.621
RN/NI	25.6	19.8	2.89	0.061

### Effect on energy metabolism and methane emission

No significant difference was observed in GE intake or urine energy output between the two treatments (Table 4). However, feeding red yeast rice significantly increased fecal energy output (P = 0.001) and decreased energy digestibility (P = 0.001) and metabolizability (P = 0.018). Feeding red yeast rice also significantly reduced CH_4_ energy output and heat production (P < 0.001), thus resulting in a higher energy retention (P < 0.001). Therefore, feeding red yeast rice produced a lower proportion of consumed ME used as heat production and a higher proportion stored in body tissue (P < 0.001).

**Table 4:** The effect of replacement of ordinary rice with red yeast rice on energy metabolism in goats (n = 20).

	CT	RYR	SED	*P*
Energy intake and output (MJ/d)				
Gross energy intake (GE)	18.2	18.3	0.12	0.763
Fecal energy (FE)	7.4	7.9	0.10	0.001
Urine energy (UE)	0.5	0.9	0.03	0.606
CH_4_ energy	1.3	1.2	0.02	<0.001
Heat production (HP)	6.1	4.9	0.13	<0.001
Digestible energy (DE) intake	10.8	10.4	0.13	0.007
Metabolizable energy (ME) intake	9.1	8.7	0.14	0.047
Retained energy (RE)	3.0	3.8	0.15	<0.001
Energy utilization (%)				
DE/GE	59.5	57.0	0.52	0.001
ME/GE	49.7	47.9	0.63	0.018
ME/DE	83.6	84.0	0.44	0.310
HP/ME	67.4	56.4	1.46	<0.001
RE/ME	32.7	43.6	1.46	<0.001

Goats fed the red yeast rice diet produced less CH_4_ (g/d) than those given the control diet and consequently had lower CH_4_ emission rates as a proportion of DM intake and OM intake (Table 5). Similar results were also obtained in terms of CH_4_ energy output as a proportion of GE intake (P < 0.001), DE intake (P = 0.004) and ME intake (P = 0.008).

**Table 5:** The effect of replacement of ordinary rice with red yeast rice on enteric methane emission in goats (n = 20).

	CT	RYR	SED	*P*
CH_4_ (g/d)	21.9	19.9	0.27	<0.001
CH_4_/Dry matter intake (g/kg)	20.3	18.3	0.29	<0.001
CH_4_/Organic matter intake (g/kg)	21.4	19.4	0.34	0.001
CH_4_ energy /Gross energy (%)	7.2	6.5	0.09	<0.001
CH_4_ energy /Digestible energy (%)	12.2	11.4	0.21	0.004
CH_4_ energy /Metabolizable energy (%)	14.6	13.5	0.30	0.008

### Effect on serum lipids and rumen fermentation variables

Feeding red yeast rice decreased the content of serum TC, TG, LDL-C and HDL-C by 47.5%, 16.0%, 45.0%, 30.3%, respectively, compared with the control (Table 6). Feeding red yeast rice had no significant effect on rumen fermentation variables in terms of rumen content of total SCFAs, acetic acid, propionic acid, butyric acid or ammonia-N, but significantly lowered the ratio of acetic acid to propionic acid (P = 0.008).

**Table 6:** The effect of replacement of ordinary rice with red yeast rice on serum lipids and rumen fermentation in goats (n = 20).

	CT	RYR	SED	*P*
Serum variables (mmol/L)				
Total cholesterol	1.77	0.93	0.31	0.019
Triglycerides	0.25	0.21	0.01	0.026
HDL- Cholesterol	0.96	0.66	0.04	<0.001
LDL- Cholesterol	0.40	0.22	0.02	<0.001
Rumen fermentation variables (mmol/L)				
Total short chain fatty acid	87.9	72.5	9.83	0.151
Acetic acid	68.4	55.1	7.20	0.102
Propionic acid	12.2	11.3	1.82	0.638
Butyric acid	7.2	6.0	1.00	0.253
Ammonia-N	8.0	8.0	0.44	0.952
Acetic/propionic acid	5.8	4.9	0.25	0.008

### Effect on rumen archaeal structure and composition

#### Sequence and alpha diversity

**Fig 1:**
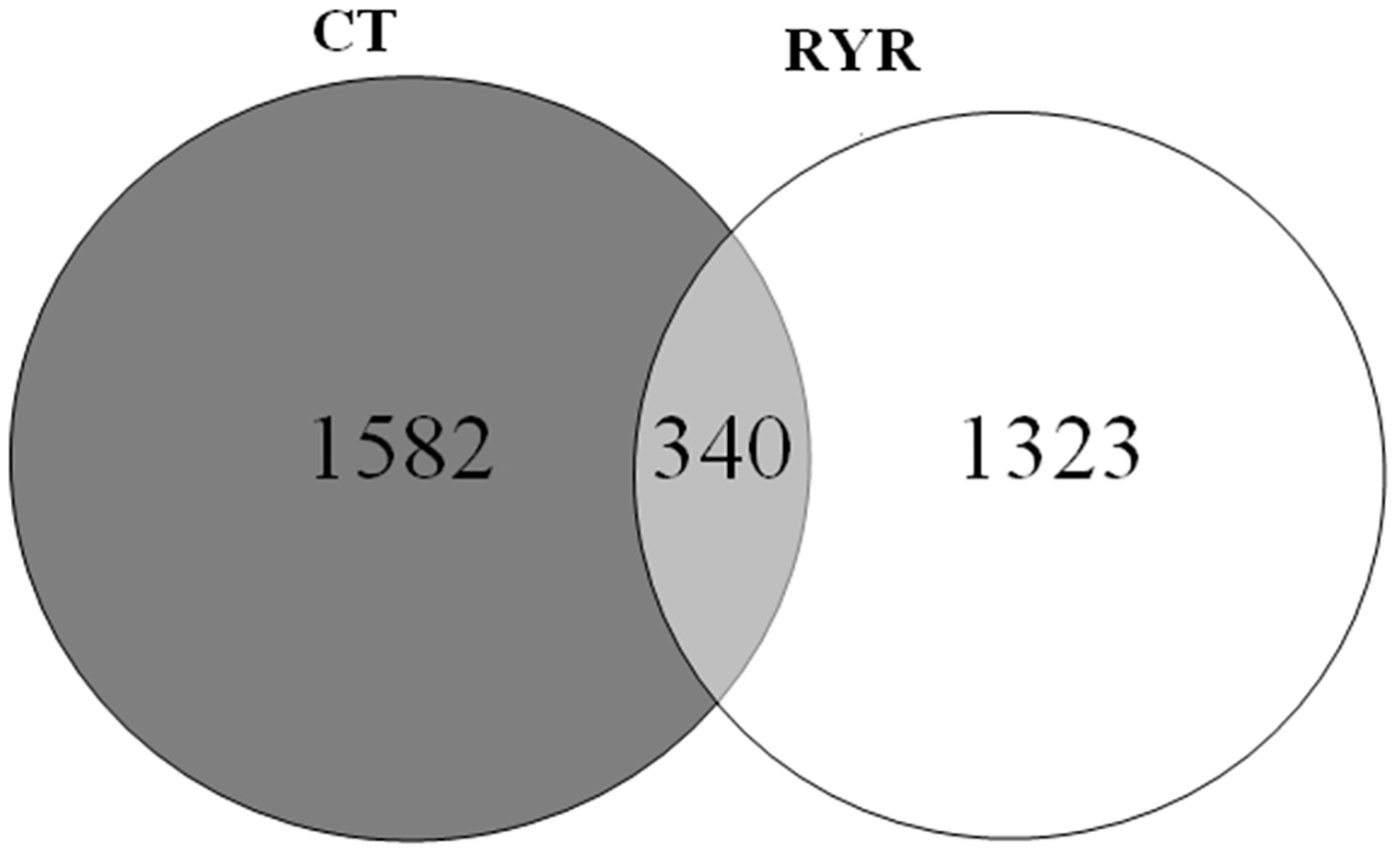
Venn plot showing the shared and unique Operational Taxonomic Units (OTUs) found in RYR group and CT group (n = 20).

In total, 75616 archaeal sequences were generated after size filtering, quality control and chimera removal. The sequences were clustered into 2364 OTUs based on 97% sequence similarity. The number of OTU in CT and RYR were 1922 and 1663, respectively, with 340 OTUs existed in all samples (Fig 1). The average OTUs between CT and RYR (172±32 vs. 146±35) were similar. Alpha diversity measures (Shannon-Wiener; the observed species; chao1) at a depth of 720 sequences are presented in Table 7. The Chao1 index and the Observed-species index did not differ significantly between the treatments. The Shannon-Wiener index reflecting the microbial richness and evenness was significantly higher (P = 0.035) in the diet RYR than in diet CT.

**Table 7:** The effect of replacement of ordinary rice with red yeast rice on alpha diversity of archaeal communities at a depth of 720 sequences in goats (n = 20).

	CT	RYR	SED	*P*
Chao1	141.7	146.7	13.6	0.722
Observed species	84.3	95.8	8.6	0.218
Shannon-Wiener	4.0	5.1	0.45	0.035

#### Archaea composition

After classification and statistics, all archaeal sequences were assigned into 3 phyla, 6 classes, 8 orders, 10 families and 11 genera. The abundances of taxa were compared from level of phylum to level of genus and the variables that were significantly different between treatments are presented in Table 8. When examining all levels from phylum to genus, in comparison with CT, RYR had significantly lower abundances for *Euryarchaeota* (P = 0.002), *Methanobacteria* (P < 0.001), *Methanobacteriales* (P < 0.001), *Methanobacteriaceae* (P < 0.001), *Methanobrevibacter* (P < 0.001) and unclassified *Methanobacteriaceae* (P = 0.007). However, RYR had significantly higher abundances for the following variables: *Crenarchaeota* (P = 0.002) and unclassified archaea (P = 0.025) (Phylum level), *Thermoprotei* (P = 0.002) and *Methanomicrobia* (P = 0.015) (Class level), unclassified *Thermoprotei* (P = 0.002) and *Methanomicrobiales* (P = 0.015) (Order level), *Methanomicrobiaceae* (P = 0.015) and unclassified *Thermoplasmatales* (P = 0.026) (Family level), and *Methanomicrobium* (P = 0.015) (Genus level).

**Table 8:** The comparison of archaeal abundances from level phylum to level genus in goat rumen between RYR group and CT group (% of sequences)^a^ (n = 20).

	CT	RYR	SED	*P*
Phylum				
*Crenarchaeota*	4.0	19.4	3.62	0.002
*Euryarchaeota*	95.8	78.7	4.11	0.002
Unclassified Archaea	0.2	1.9	0.6	0.025
Class				
*Thermoprotei*	4.0	19.4	3.62	0.002
*Methanobacteria*	64.0	32.6	4.42	<0.001
*Methanomicrobia*	2.7	8.5	2.00	0.017
Order				
Unclassified *Thermoprotei*	3.9	19.1	3.61	0.002
*Methanobacteriales*	64.0	32.6	4.42	<0.001
*Methanomicrobiales*	2.1	8.2	2.04	0.015
Family				
*Methanobacteriaceae*	64.0	32.6	4.42	<0.001
*Methanomicrobiaceae*	2.1	8.1	2.02	0.015
Unclassified *Thermoplasmatales*	3.6	7.8	1.60	0.026
Genus				
*Methanobrevibacter*	63.8	32.3	4.49	<0.001
Unclassified *Methanobacteriaceae*	0.2	0.0	0.04	0.007
*Methanomicrobium*	2.1	8.1	2.02	0.015

^a^Only the taxa that were significantly different (P < 0.05) between treatments were presented.

#### Cluster analysis

**Fig 2:**
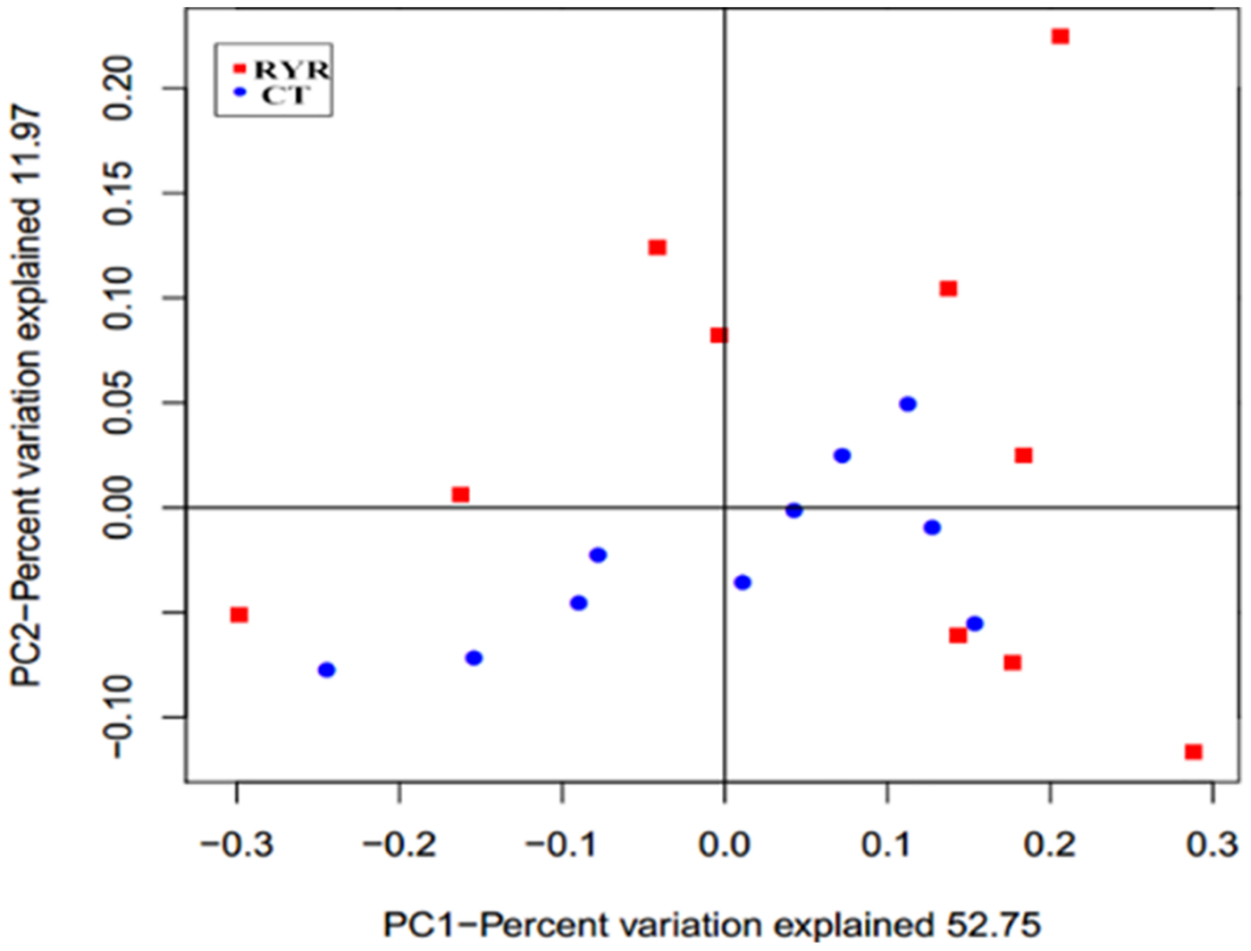
Principal Co-ordinate Analysis (PCoA) of archaeal Operational Taxonomic Units from goat rumen fluid.

The weighted uniFrac distance matrix within samples was measured according to the OTU table. The similarity of samples in RYR and in CT and between RYR and CT were 65.0%, 70.6% and 62.8%, respectively. The result indicated the samples in CT were more similar than in RYR. The cluster of samples was visualized using PCoA (Fig 2). It was clear that the samples in RYR were more scattered than those in CT.

#### Shared genus

A genus-level analysis was performed to identify the core archaeal community which was shared by the samples of CT, RYR and all samples (Table 9). There were nine genera and eight genera shared by CT and RYR, respectively, and seven genera were shared by all goats. The most abundant genus shared by CT, RYR and all samples was *Methanobrevibacter*, followed by *Thermogymnomonas*. *Methanosphaera* and *Methanimicrococcus* were shared only within CT.

**Table 9:** The archaeal genera shared by CT group (n = 10), RYR group (n = 10) and all goats (n = 20) and their abundance (Means ± SD, % of sequences)^a^.

Genus	CT	RYR	All animals
(Order) Unclassified *Thermoprotei*	3.9±2.8	19.1±11.8	11.5±11.4
*Methanobrevibacter*	63.8±15.7	32.4±15.3	48.05±22.1
*Methanosphaera*	0.1±0.1	/^b^	/
*Methanomicrobium*	2.1±1.7	8.1±6.4	5.1±5.5
*Methanimicrococcus*	0.6±0.9	/	/
(Family)Unclassified *Thermoplasmatales*	5.5±7.4	3.5±3.8	4.5±5.8
*Thermogymnomonas*	17.6±9.2	23.3±12.9	20.5±11.3
(Family)unclassified *Thermoplasmatales*	3.6±4.1	7.8±5.9	5.7±5.4
(Class)unclassified *Euryarchaeota*	2.4±1.5	3.0±3.8	2.7±2.8
(phylum)unclassified Archaea	/	1.9±2.2	/

^a^Taxa that could not be assigned to a genus but still shared by the samples were displayed using the highest taxonomic level that could be assigned to them and the level is shown in parentheses.

^b^ “/”means this item did not exist in this column.

## Discussion

Before the commencement of the present study, we conducted a range of preliminary trials. In these trials, different rates of red yeast rice (4%, 5%, 6%, 7%, 8%, DM basis) were included in diets of goats, with feed intake and enteric methane emissions determined using indirect open-circuit respiration chambers. The results of these preliminary trials showed that the including rates less than 8% had no significant effects on enteric methane emissions per kg DM intake (S1 Table). The inclusion rate of 8.2% (DM basis) used in the present study was determined according to lovastatin concentration in red yeast rice (2 mg/g DM, inactive L-form of 44%) and the results of these preliminary trials. Each goat was fed a restricted amount of diet at 1.1 kg/d (DM) in the present study, giving a maximum ingestion of L-form lovastatin at 80 mg/d, which is the maximum dose recommended for a human adult [24]. Therefore, only one rate of red yeast rice in diet was used in the present study.

In the present study, feeding red yeast rice significantly reduced TC, TG, HDL-C and LDL-C concentrations in serum. The reason for lowering the lipid concentration is that lovastatin contained in red yeast rice can inhibit the biosynthesis of cholesterol. Many previous studies demonstrated the potential of red yeast rice for lowering lipid concentration in serum. For example, research in human conducted by Wang *et al*. [25] showed that TC, LDL-C and HDL-C concentrations in serum decreased by 22.7%, 30.9% and 34.1%, respectively, after 8 weeks of red yeast rice treatment. Wei *et al*. [26] reported that total cholesterol content in serum was 25% or 40% lower in rabbits fed 0.4 or 1.35 g/kg/day of red yeast rice when compared to control rabbits.

Although ADF and NDF digestibility were similar between the two treatments, feeding red yeast rice significantly reduced organic matter, energy and nitrogen digestibility in the present study. The mechanism by which this occurs is unclear at present. It may be related to the high content of lovastatin in the diet. In the present study, the total ingestion of lovastatin each goat reached up to 180 mg/d and 4.3 mg/kg live weight each day. Although lovastatin are considered well tolerated in human, many side effects such as anaphylaxis [27], toxic hepatitis [28] and rhabdomyolysis [29], etc. had been reported, especially in case of overdose. Ramírez-Restrepo et al. [10] also reported the onset of adverse effects in terms of DMI and animal physiology when cattles were fed 2.6 mg/kg live weight for 4 days. Red yeast rice also contains many non-statin secondary bioactive components such as citrinin. Citrinin is a mycotoxin which has the potential to cause adverse effect such as liver and kidney injury if large amount of it was absorbed into body [30], so then probably leads to the decline in the utilization of nutrients. Since we did not detect the content of the citrinin in the diet, we could not determine whether the decrease in the digestibility in the present study was associated with it. It was worth noting that, although the digestibility of energy was decreased, the supplement of red yeast rice significantly increased the retained energy (MJ/d). This should be attributed to the mitigation of CH_4_ emission and thus the decrease of heat production.

The results in the present study indicated a decrease of 13% in CH_4_/DM intake (g/kg) when goats were fed on red yeast rice -containing diets. Up to date, evaluation of the effect of lovastatin on enteric CH_4_ emission has been concentrated on using *in vitro* techniques, and there were only a few *in vivo* publications. Miller and Wolin [5] in an *in vitro* study found that HMG-CoA reductase inhibitors (lovastatin and mevastatin) had ability to inhibit methanogens growth, and addition of lovastatin at 4 nmol/ml resulted in 50% decreased in the growth of a strain of *Methanobrevibacter*, and 10 nmol/ml could completely stop methanogens growth and CH_4_ production [6]. Soliva *et al*. [31] using a rumen simulation technique (Rusitec) reported that a decrease in CH_4_ production by 42% at a supplementation rate of 150 mg lovastatin per liter of rumen liquor. Faseleh *et al*. [7] found that lovastatin had ability to block cell membrane synthesis in methanogens and that 75% of total lovastatin in fermented rice straw extracts was in the active hydroxyacid form, which had a stronger effect in reducing both growth and CH_4_ production in *Methanobrevibacter* when compared to commercial lovastatin. However, it is worth noting that doses of lovastatin used in *in vitro* experiments were very high, and its effect needs to be verified with *in vivo* trials. On the other hand, Klevenhusen et al. [32] conducted an *in vivo* experiment in sheep by feeding lovastatin at 93 mg/d and failed to observe any effect on total daily CH_4_ production.

The difference between *in vivo* and *in vitro* studies probably reflects high doses used *in vitro* studies. The concentration of lovastatin *in vitro* study of Soliva *et al*. [31] was equivalent to 10 g/kg DM, which was 100 times higher than that (80 mg/kg DM) used in the study of Klevenhusen *et al*. [32]. It was undesirable to add such high doses of lovastatin in animal diets. The reason lies in that lovastatin would deplete large amount of esterase when it is absorbed into body, which would harm human/animal health. Therefore, it is recommended that the maximum dose of lovastatin ingested by an adult human should not exceed 80 mg/d [24]. Furthermore, lovastatin is a prescribed medicine used as cholesterol inhibitor in human, thus it is impossible to be administered as an animal feed additive. The red yeast rice contains natural lovastatin with a relatively high percentage of active H-form. Hence, theoretically red yeast rice is an appropriate replacement in inhibiting archaeal growth in the rumen of ruminant animals. A previous trial found that feeding red yeast rice to sheep reduced enteric CH_4_ emission by 30% [9], which agreed with our result in goats, although Ramírez *et al*. [10] reported that inclusion of red yeast rice in cattle diets only produced a transient reduction in CH_4_. The disagreement probably was caused by variation of different animal species used and/or different types of red yeast rice supplemented. The composition and content of bioactive component in red yeast rice vary depending on the fermentation conditions of rice and *Monascus* strains used [7]. Further research is required to explore how to increase lovastatin content and active H-form proportion, thus enhancing the effect of red yeast rice in mitigating CH_4_ emission of cattle, sheep and goats.

Methanogens and other archaea have unique membrane lipids that contain glycerol joined by ether linkages to long chain isoprenoid alcohols [33]. Synthesis of isoprenoid units needs a key precursor, mevalonate, which is formed by reduction of HMG-CoA. The HMG-CoA reductase inhibitors have the potential to specifically inhibit archaea in the rumen [5], and do not have any negative effect on rumen fermentative bacteria because their lipids are glycerol esters of long chain fatty acids. This had been proved by Miller and Wolin with an *in vitro* study [6], in which lovastatin did not inhibit the growth of species of rumen bacteria that are essential for fermenting cellulose, starch and other plant polysaccharides. Faseleh *et al*. [8] found that fermented rice straw extracts could reduce total methanogens population and increase cellulolytic bacteria including *Ruminococcus albus*, *Fibrobacter succinogenes* and *Ruminococcus flavefaciens*. Our results showed that, at the genus level, the predominant archaea were *Methanobrevibacter* (63.8%) in the rumen of control goats, which was in agreement with many previous studies [34–40]. In the present study, we noticed that the abundance of *Methanobrevibacter* was significantly decreased by the supplementation with red yeast rice in goats. Previously, using *in vitro* studies, Miller and Wolin [5, 6] and Faseleh *et al*. [7] reported that lovastatin inhibited the growth of *Methanobrevibacter*. The present study was the first *in vivo* report about red yeast rice inhibiting the growth of *Methanobrevibacter*. Previous studies indicated that, except archaea, other microorganisms in the rumen including bacteria, fungi and protozoa, did not have HMG-CoA reductase. Evidences indicated that many archaeabacreria including *Haloferax volcanii* [41], *Sulfolobus solfataricus* [42], *Methanococcus jannaschii* [43] have gene coding HMG-CoA reductase in their DNA. Therefore, it is deduced in theory that the inhibitor of HMG-CoA reductase may have potential to inhibit all archaea in the rumen. Nevertheless, in the present study, the abundance of some archaea kept stable and some (such as *Methanomicrobium*) even increased by the treatment, although the abundance of *Methanobrevibacte* decreased by nearly 50% with feeding red yeast rice. This phenomenon probably suggested that part but all of archaeabacteria need the participation of HMG-CoA reductase in their growth and proliferation. This deserves to be further explored with *in vivo* experiments.

## Conclusions

In the present study, feeding goats with red yeast rice had not negative effect on fibre digestion and rumen fermentation. The red yeast rice treatment significantly reduced enteric CH_4_ emissions, serum lipid and growth of some archaea species (e.g., *Methanobrevibacter*) in the rumen. Therefore, as a natural fermentation product, red yeast rice has potential to reduce enteric methane emissions of goats. However, caution should be taken when it is used because it may inhibit the digestibility of protein and organic matter. Further studies are required to evaluate its potential with different diets and animal species and its effects on animal health and food safety.

## Supporting Information

S1 Table The results of the preliminary trials. The effect of dietary replacement of ordinary rice with different rates of red yeast rice on enteric methane emission in goats (n = 36).
(DOCX)
