# Supplementary material for: The Effect of Dietary Replacement of Ordinary Rice with Red Yeast Rice on Nutrient Utilization, Enteric Methane Emission and Rumen Archaeal Diversity in Goats
Source: PLoS One. 2016 Jul 28;11(7):e0160198. doi: 10.1371/journal.pone.0160198 (PMC4965012; doi:10.1371/journal.pone.0160198)
Supplement: S1 Table — The effect of dietary replacement of ordinary rice with different rates of red yeast rice on enteric methane emission in goats (n = 36). (DOCX) [file pone.0160198.s001.docx]

**S1 Table. The results of the preliminary trials.** The effect of dietary replacement of ordinary rice with different rates of red yeast rice on enteric methane emission in goats (n=36).

| Treatment | CH_4_ emission/dry matter intake (g/kg)* |
| --- | --- |
| Control | 20.3±1.1^a^ |
| 4% group | 20.4±1.0^a^ |
| 5% group | 20.5±0.7^a^ |
| 6% group | 20.7±1.0^a^ |
| 7% group | 20.2±0.9^a^ |
| 8% group | 18.2±1.1^b^ |

* In the same column, values with different superscripts mean significant difference (P<0.05).
